# Editorial: Optimizing athletic recovery: the effects of recovery strategies and sleep on sports performance

**DOI:** 10.3389/fspor.2026.1848719

**Published:** 2026-04-17

**Authors:** Rui Miguel Silva, Francisco Tomás González-Fernández, Rafael Oliveira, Rodrigo Aquino

**Affiliations:** 1 Research Center in Sports Performance, Recreation, Innovation and Technology (SPRINT), Melgaço, Portugal; 2 Escola Superior Desporto e Lazer, Instituto Politécnico de Viana do Castelo, Rua Escola Industrial e Comercial de Nun’Álvares, Viana do Castelo, Portugal; 3 Combat Sports Psychology Lab, Los Angeles, CA, United States; 4 Department of Physical Education and Sports, Faculty of Sport Sciences, Sport and Health University Research Institute (iMUDS), University of Granada, Granada, Spain; 5 CTS-1172: Group on Innovative Strategies for Advancing Performance, Psychology, Education and Kinesiology Granada, Spain; 6 Research Center in Sports Sciences, Health Sciences and Human Development (CIDESD), Santarém Polytechnic University, School of Sport, Rio Maior, Portugal; 7 Santarém Polytechnic University, School of Sport, Rio Maior, Portugal; 8 Federal University of Espírito Santo, Vitória, Brazil

**Keywords:** athlete monitoring, exercise physiology, exercise testing, recovery strategies, sleep

## Introduction

In contemporary sport, recovery has emerged as a central component of the training and competitive process. It is no longer regarded simply as the time that follows exercise or competition, but rather as a complex and multidimensional process that supports physiological restoration, psychological readiness, adaptation to training, and the preservation of performance over time. As the physical and mental demands placed on athletes continue to increase, the capacity to recover effectively between sessions and competitions has become a matter of considerable importance for coaches, practitioners, and researchers alike. Growing interest in sleep, well-being, and recovery-related interventions reflects a broader recognition that successful performance depends not only on the quality of training but also on the quality of restoration that follows it.

This Research Topic was conceived in response to that need. It sought to explore the interplay between recovery strategies, sleep, and sports performance, while also considering the physiological, psychological, and practical dimensions through which recovery may be understood and optimized. In doing so, the topic encouraged contributions that advanced both scientific knowledge and applied practice. Such an approach is especially timely, given that recovery in sport cannot be adequately explained through single variables or isolated mechanisms. Rather, it must be approached as a dynamic process influenced by training load, sleep quality, emotional regulation, biological readiness, and the specific demands of different sporting environments.

The five articles included in this Research Topic reflect this broad perspective. Together, they address the relationship between sleep and neuromuscular performance, the effects of high-intensity exercise on subsequent sleep, the potential influence of an applied recovery device on sleep in elite football players, the role of mindfulness in sleep and recovery stress states, and the association between antioxidant status and strength-related performance in combat athletes. Although diverse in focus, these studies converge in highlighting the multifactorial nature of recovery and the need for more integrative approaches to athlete support.

## Summary of the included articles

Sleep is widely acknowledged as one of the most important pillars of athletic recovery, yet the specific aspects of sleep that most strongly relate to performance remain an area of ongoing investigation. In the study by Cabarkapa et al. the authors examined the association between sleep quality, sleep quantity, and countermovement jump force time characteristics in semi-professional basketball players. Their findings indicated that sleep quality appeared to be more closely associated with lower body neuromuscular performance than sleep quantity, particularly in variables related to concentric force and power production. Sleep quantity, by contrast, was not significantly associated with the assessed outcomes. These findings are noteworthy because they suggest that sleep quality may represent a more meaningful indicator of recovery status than duration alone, particularly when considering performance capacities that are central to basketball. Nonetheless, this study was made in male basketball players with an average of 7.9 h of sleep which means that other athletic populations with different sleep quantities may present different results.

The relationship between exercise characteristics and sleep responses was explored further in the study by Kong et al. In this investigation, adolescent speed skaters completed different forms of high-intensity exercise, including aerobic capacity testing, Wingate testing, and interval training, in order to compare their effects on subsequent sleep quality. The authors reported that both Wingate testing and interval training negatively influenced sleep, producing shorter sleep duration, greater physiological stress during sleep, lower RMSSD, and a higher LF to HF ratio. Aerobic capacity testing did not appear to impair sleep in the same way. These findings underline the importance of exercise modality and intensity in shaping recovery responses and suggest that certain forms of intense anaerobic exercise may interfere with one of the most important mechanisms of overnight restoration.

A particularly applied contribution to the topic was offered by study Moen et al. This study examined whether Bio-Electromagnetic Energy Regulation therapy influenced subjective sleep in elite female football players over an extended period of monitoring. The authors found that both subjective sleep duration and sleep quality declined on game nights, a result that is consistent with the broader literature showing that competition may disrupt sleep at precisely the time when recovery is most needed. Importantly, the study also observed improvements in subjective sleep measures during the intervention period compared with the preceding control period. Although the results should be interpreted with due caution since only 21 elite female football were analysed, the study contributes meaningfully to the field by addressing a practical recovery strategy within a real elite sport context, thereby helping to bridge the gap between research and application. It is relevant to mention that menstrual cycle can also influence sleep quality which was not analysed [See Rugvedh et al. (1) for more information].

The psychological dimension of recovery was brought into focus by Birnkraut et al. study. Using a continuous two-week monitoring design in elite judoka, the authors investigated how both trait mindfulness and day-to-day fluctuations in mindfulness related to sleep and recovery stress states. Their findings demonstrated positive associations between mindfulness and qualitative subjective sleep parameters, as well as morning and evening recovery-related states. Among the different mindfulness facets, acting with awareness emerged as the most influential predictor. The study also showed that mindfulness was related to subjective and objective sleep latencies, suggesting that mental regulation may play a relevant role in the transition to sleep. These findings are important because they extend the concept of recovery beyond purely physiological parameters and indicate that psychological skills and attentional processes may meaningfully shape how athletes rest, recover, and prepare for subsequent demands.

The final article in the collection, by Huang et al. broadened the discussion by examining the relationship between antioxidant-related indicators and strength-related performance in combat athletes. The authors reported that ATP was positively associated with standing long jump and vertical jump performance, whereas body fat percentage was negatively associated with vertical jump. They also identified differences in body fat percentage and superoxide dismutase activity across sports, as well as lower total antioxidant capacity values in female athletes compared with males. Although the regression models did not achieve statistical significance and the findings were appropriately presented as exploratory, the study offers an important contribution by linking performance-related outcomes with biological and metabolic characteristics that may have implications for readiness, recovery, and longer-term athlete support.

The articles included in this Research Topic provide a rich and multifaceted perspective on athletic recovery. They show that recovery is not determined solely by the amount of rest that follows exercise or competition, but also by the quality of sleep, the nature of the training stimulus, the psychological resources available to the athlete, and the internal physiological state that supports performance and adaptation. Across sports as varied as basketball, speed skating, football, judo, and combat disciplines, the studies collectively reinforce the view that recovery must be understood as an integrated and context-dependent process. This collection also points to several important directions for future research. There remains a clear need for more longitudinal and experimental work capable of clarifying causal pathways and establishing the practical efficacy of different recovery interventions. Greater integration of subjective and objective monitoring methods would further strengthen the ecological validity of future investigations, while more individualized approaches are needed to account for the substantial variability that exists between athletes in their responses to training, competition, sleep disruption, and recovery strategies. The replication of the studies included in the Research Topic in other sports will strengthen and potentially confirm the present findings. Advancing in the field in these ways is essential if recovery science is to move toward more precise, evidence-informed, and sport-specific recommendations.

This Research Topic contributes to the expanding literature that recognizes athletic recovery as a complex and integrated process with important implications for both performance and well-being. Considering these studies that examine sleep, training stress, psychological functioning, applied interventions, and biological markers, this Research Topic collection increases the current understanding of recovery complexities for both researchers and practitioners and reinforces previous suggestions which pointed that poor sleep affects (soccer) players' performance and increases the risk of injury (2). It is hoped that the work presented here will stimulate further interdisciplinary inquiry and encourage the continued development of effective strategies to support recovery, health, and sustained performance across a wide range of sporting contexts.

## References

[1] Rugvedh P Gundreddy P Wandile B. The menstrual cycle’s influence on sleep duration and cardiovascular health: a comprehensive review. Cureus. (2023) 15(10):e47292. 10.7759/cureus.47292 38022155 PMC10656370

[2] Clemente FM Afonso J Costa J Oliveira R Pino-Ortega J Rico-González M. Relationships between sleep, athletic and match performance, training load, and injuries: a systematic review of soccer players. Healthcare. (2021) 9(7):808. 10.3390/healthcare9070808 34206948 PMC8305909

